# Supplementary material for: Complexome profiling of the Chlamydomonas psb28 mutant reveals TEF5 as an early PSII assembly factor
Source: Plant Cell. 2025 Mar 17;37(6):koaf055. doi: 10.1093/plcell/koaf055 (PMC12164590; doi:10.1093/plcell/koaf055)
Supplement: koaf055_Supplementary_Data [file koaf055_supplementary_data.zip › psb28_interactive.zip!/plots/intensity/qualityPlots/peptides/VLE1_Cre01.g049950.t1.1.html]

### Cre01.g049950.t1.1

Trivial name: VLE1  
SeqCoverage [median]: 13.80  
Mass: 123.22 kDa  
  
 

| Sequence | MissedCleavage | Mass [Da] | wtIntensity | muIntensity | Score |
| --- | --- | --- | --- | --- | --- |
| AFNLPGVHYQTLAGAR | 0 | 1713.90 | 1116.54 | 10707.79 | 53.83 |
| AFSQGVAFR | 0 | 981.50 | 837.76 | 4089.61 | 102.72 |
| AIDYGYAK | 0 | 899.44 | 2039.62 | 2343.33 | 94.41 |
| ATPQTPLQIK | 0 | 1095.63 | 1560.35 | 1544.51 | 74.13 |
| FFYSPNPNTVASLPR | 0 | 1708.86 | 917.28 | 1742.12 | 136.53 |
| IFSMSFGQDAR | 0 | 1257.58 | 594.82 | 2251.08 | 109.66 |
| LLVTSHMR | 0 | 955.53 | 0.00 | 246.94 | 74.42 |
| LSSNMQIGGMR | 0 | 1192.57 | 364.68 | 0.00 | 60.40 |
| MGTIMR | 0 | 707.35 | 1696.62 | 600.82 | 58.25 |
| QAEDPTLSLYK | 0 | 1263.63 | 1148.75 | 0.00 | 84.82 |
| TSATPTNKPSLDAAATAYR | 1 | 1934.97 | 2184.05 | 3586.54 | 117.84 |
|  |  |  | 12460.5 | 27112.7 |  |
